# Report on Dental Education

**Published:** 1874-08

**Authors:** E. S. Holmes

**Affiliations:** Committee


### REPORT ON DENTAL EDUCATION.

*Read before the Michigan Dental Society, by a Special Committee, at the Annual Meeting,* 1872.

MR PRESIDENT AND GENTLEMEN OF THE MICHHGAN DENTAL ASSOCIATION.

From remarks made at the session of this association, at Grand Rapids, in 1871, relative to the practice of dental colleges in graduating persons who had not taken a regular tuition, and been subjected to an examination, your committee thought it advisable to investigate the matter; and to this end, the following note was written to the deans of the faculties of each of the dental schools in the United States; viz: To the Baltimore College of Dental Surgery; the Ohio College of Dental Surgery the; Pennsylvania College of Dental Surgery; Philadelphia Dental College; Missouri Dental College; the Saint Louis Dental College; the New Orleans Dental College; the New York College of Dentistry; the Boston, Dental College; and to the Harvard University Dental Department.

NOTE.

"Dear Sir: At the last meeting of the Michigan Dental Association the value of the diplomas of dental colleges was under animadversion. Some contended that a diploma could be bought of any dental college, and, therefore, its possession was no evidence of the professional qualifications of its possessor.

Now will you be kind enough to inform me at your earliest convenience, whether or not the college in which you are a Professor, and of which you are the Dean, does at any time, and under any circumstances grant diplomas to any persons, except upon full evidence of their qualifications to practice dentistry, by or after an examination. And if so, under what circumstances do you grant such diplomas? I refer to present practice, say within the last five years, and to the future.

Your immediate attention to this will much oblige our association, and your well wisher." E. S. Holmes.

*Member of Committee on Dental Education, Mich. Dental Association.*"

We proposed to limit the investigation to the "present practice" of the dental schools, because there was some excuse for the older colleges in their earlier history to confer honorary degrees upon dentists known to be worthy by reputation, for the purpose of interesting them in the cause of a collegiate education, to induce them to send young men to these institutions and to advertise them. But we consider that there is no such excuse at present; as the existence of such institutions of learning is known to every one interested in the subject, and the facilities for obtaining a thorough education in dentistry are as great and as easily accessible as in any other profession.

To the above "Note" we have received the following replies; which form part of our report, viz : From Harvard University Dental Department; Boston Dental College ; New York College of Dentistry ; Missonri Dental College ; Pennsylvania College of Dental Surgery; Ohio College of Dental Surgery.

From these replies it would seem that the public have nothing to fear, and may reasonably entertain very high expectations from the professional practice of the graduates of these schools. It is truly gratifying to see a determination to supply the demand for higher scholastic attainments, as well as manipulative ability in the coming dentist. It is reasonable to expect that these schools, at least, will from, year to year, send out graduates who will bless mankind and be an honor to the profession of their choice.

We can only say with reference to those collges from whose officers we have received no replies to our friendly note—that we know nothing about them. We certainly could not advise any one desirous of obtaining a thorough dental education to apply to any of them for this purpose. Their silence would seem to indicate that they are guilty of the practice charged generally to all dental colleges at the last year's session. But as we have nothing positive against them we would suggest that this investigation be continued, hoping that they will all be able to prove themselves entirely above suspicion.

It is probable that some of the dental colleges are injudicious in occasionally conferring degress upon unworthy applicants, yet it is unquestionably true that the great majority of the graduates from the dental schools of this country are entirely worthy of their professional title. Your committee would therefore respectfully recommend that this association as a body, and its members as individuals, do all in their power to correct the errors, and maintain the excellences of our dental colleges. They are certainly the most potent of all the means now made use of for the promotion of dentistry to the position it ought to occupy as a learned profession. Let us as tutors, send to our colleges better material—more advanced and cultivated minds—then our students, and their graduates will be, at least, proportionally, increased in excellence ; and attain and maintain a much higher professional standing. It is not sufficient for us to take young men into our offices as students for two years, or for three years; and all this time keep them at work at a mechanical trade—the manufacture of artificial dentures—and then expect that our colleges will make dentists of them—worthy the name—in two short courses of lectures. Our students must have higher scholastic attainments before they become dental students, and then more thorough professional and scientific training in our offices, then our colleges will send out graduates that will not deceive or disappoint, but bless their patrons, and honor our profession.

SUPPLEMENTARY REPORT OF THE COMMITTEE ON DENTAL EDUCATION.

*Read before Michigan Dental Association*, 1873.

In the report of your "Committee on Dental Education" made at our last meeting, it was stated that on account of dental colleges having been accused of certain loose practices in the matter of graduating persons for money, they had written to the dean of each of the dental schools asking for information on this subject. Answers were received from the following schools, averring that they did not and would not on any consideration graduate any person, except according to, and in fulfilment of their published terms of graduation, viz: Missouri Dental College; Dental Department of Harvard University ; Boston Dental College ; Pennsylvania College of Dental Surgery ; New York College of Dentistry.

After the committee had made their report, and just before the close of the session, a member presented a letter from Prof. Taft, of the Ohio College, of Dental Surgery, from which we understand that whatever may have been the practice of this school in the earlier days of its existence, in the future they would adhere strictly to their published terms of graduation.

Since the last meeting of this association, we have received answers from the following colleges which we will read as a part of this report, viz:

Philadelphia Dental College; Baltimore College of Dental Surgery; New Orleans Dental College. These letters speak for themsevles; and express the sentiments that every progressive dentist is glad to hear.

The Saint Louis Dental College has not answered our letter, and from the fact that the faculty of that concern have never done anything to promote the true education of dentists, so far as your committee can ascertain, and have embraced such opportunities as offered to sell sheep-skins, we are not at all disappointed at their silence.

Another, the Maryland, has just, this year, started on what we hope will be a successful career of usefulness. As the charges made against dental colleges two years ago could not apply to this school, its faculty have not been questioned on the subject.

From the evidence presented last year, and, now your committee is happy to be able to state that the sweeping assertion made in a paper read before this association two years ago, was entirely without foundation. In the earlier days of the older dental schools it was thought advisable to graduate a few old dentists who were known to be capable and reputable practitioners, without requiring them to conform to the curriculum. Such action at that time was, perhaps, wise. But the reasons for pursuing that course have passed away; and it is gratifying to know that the colleges understand it as well as we do, and have determined in the future to adhere strictly to their published rules in granting diplomas. Therefore, we may safely calculate that a future graduate of a dental college will possess a good character, a thorough education, and a good degree of professional skill.

How much the action of your committee has done to bring about this desired result, we of course cannot tell. Perhaps not anything. We fondly hope it has done no harm. It certainly will not do injury to have it distinctly understood by our dental teachers, and by the trustees of the dental colleges that they are watched, not only by dentists in general, but by dental associations in particular.

But, however much gratified we may be that the colleges are pledged to adhere strictly to their rules, we can but regret that the rules do not require a higher standard of scholarship. We are aware that the colleges labor under many difficulties. A very important one is, that the dentists of the country send to them matriculants who do not possess a proper amount of preliminary education. It is impossible in two short courses of lectures to lay a scientific foundation, and build a professional superstructure also. It would be an important step in the right direction if both medical and dental schools would refuse to matriculate any one who did not possess sufficient elemantary and scientific acquirements to be a graduate of a high school at least. In fact, we would be glad to see the time when the only avenue of access to a medical school will be through a scientific college, and when one of the requirements of the candidate for graduation by all dental colleges will be the possession of a diploma conferred by a medical college. As the dentist is expected to treat and cure the insidious diseases of the human system that pertain to his specialty. It is just as important that he should possess a thorough medical and surgical education, as that the practitioner in any other department of cure should. And in addition to this general knowledge, the dentist must receive a special medical and surgical training and acquire great manipulative or operative dexterity to fit him for his professional duties, which can only be properly acquired, in a reasonable length of time, in dental schools.

Hence the importance of confidence in dental colleges. And as the assertion made two years ago on the floor of this association by a graduate of one of the dental colleges, showed a want of confidence, your committee thought it best to investigate the subject and report. While we believe the gentleman was actuated by the best of motives, and had the good of our profession and of the colleges in mind in making the derogatory statement; the evidence shows that he was laboring under a mistake. That some, and perhaps all of our dental colleges may have graduated unworthy persons is quite possible,—for to err is human—and what graduating institution has not done so. But we feel warranted in asserting our confidence in the value of dental college diplomas,and that as a general rule—amounting almost to universality—the fact that a person possesses an unannulled diploma conferred by a legally authorized dental school is, prima facie, evidence that he is worthy of confidence as a gentlemen and as a practitioner of dentristry. It would afford us unbounded pleasure to know that every practicing dentist in Michigan possessed such evidence of ability. The people would suffer immeasurably less from empiricism and mal-practice, and would enjoy the possession of their natural organs of mastication in health and happiness.

E. S. Holmes, Committee.

